# Supplementary material for: A Global Screen for Assembly State Changes of the Mitotic Proteome by SEC-SWATH-MS
Source: Cell Syst. 2020 Feb 26;10(2):133–155.e6. doi: 10.1016/j.cels.2020.01.001 (PMC7042714; doi:10.1016/j.cels.2020.01.001)

O60566 | BUB1B_HUMAN | BUB1B BUBR1 MAD3L SSK1
Monomer MW [kDa]: 119.545 Monomer expected elution fraction: 39

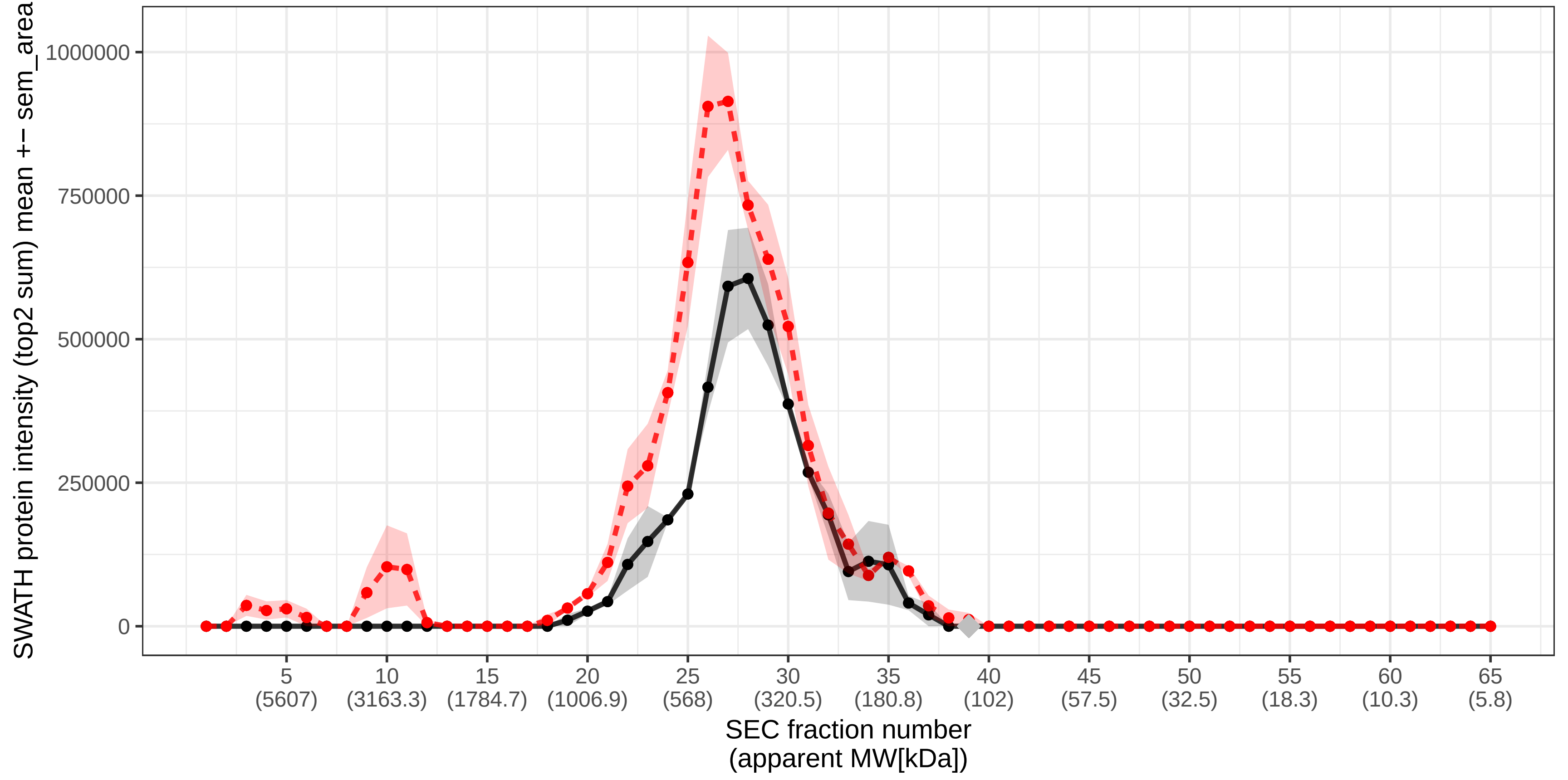

Supplement: Data S1. SEC-SWATH-MS Protein Chromatograms, Related to Figure 1 [file mmc6.zip › SECchrom_O60566_BUB1B_HUMAN_BUB1B_BUBR1_MAD3L_SSK1.pdf]
